# Parasitic Infections among Children under Five Years in Senegal: Prevalence and Effect on Anaemia and Nutritional Status

**DOI:** 10.5402/2013/272701

**Published:** 2013-12-26

**Authors:** Roger C. K. Tine, Babacar Faye, Cheikh T. Ndour, Khadime Sylla, Doudou Sow, Magatte Ndiaye, Jean L. Ndiaye, Pascal Magnussen, Michael Alifrangis, Ib C. Bygbjerg, Oumar Gaye

**Affiliations:** ^1^Université Cheikh Anta DIOP de Dakar, Faculté de Médecine, Pharmacie et Odontologie, Service de Parasitologie Médicale, BP 5005, Dakar, Senegal; ^2^Clinique des Maladies Infectieuses, Centre Hospitalier Universitaire de Fann, Senegal; ^3^Department of International Health, Immunology and Microbiology, Faculty of Health Sciences, University of Copenhagen-CSS, Øster Farimagsgade 5, 1014 Copenhagen, Denmark

## Abstract

Although malaria is declining in many countries in Africa, malaria and anaemia remain frequent in children. This study was conducted to assess the relationship between malaria parasitaemia, intestinal worms, and anaemia, in children <5 years living in low transmission area in Senegal. A survey was carried out in 30 villages in the central part of Senegal. A two-level random cluster sampling technique was used to select study participant. Children <5 years were enrolled after informed consent. For each child, blood thick and smear tests were performed, haemoglobin concentration was measured with HemoCue, and stool samples were collected and examined using the Ritchie technique. A total of 736 children were recruited. Malaria parasite prevalence was 1.5% (0.7–2.6); anaemia was found in 53.4% (48.2–58.9), while intestinal parasites and stunting represented 26.2% (22.6–30.2) and 22% (18.6–25.5), respectively. In a logistic regression analysis, anaemia was significantly associated with malaria parasitaemia (aOR= 6.3 (1.5–53.5)) and stunting (aOR = 2 (1.2–3.1)); no association was found between intestinal parasites and anaemia. Malaria and anaemia remain closely associated even when malaria is declining. Scaling up antimalarial interventions may contribute to eliminate malaria and reduce the occurrence of anaemia among children.

## 1. Introduction

Malaria is a major public health problem in African region. According to the World Health Organization (WHO), in 2010, there were an estimated 149 to 274 million cases and 537,000 to 907,000 deaths worldwide [1]. Over 89% of these deaths occur in Africa, mainly in children under 5 years, most of the time outside health facilities [2]. In recent years, significant reductions in the incidence of malaria have been reported in several countries in Africa where malaria was previously moderately endemic [3]. Although the incidence of malaria appears to be declining in a number of African countries, malaria remains an important cause of mortality and morbidity among young children.

Anaemia also remains a major public health problem, not least in malaria endemic areas, where it is primarily seen in young children in areas with stable malaria, but also in adults in malaria unstable areas [4, 5]. Recently, the WHO advocated for the scaling up of malaria control interventions in order to accelerate malaria elimination and consequently reduce malaria fatal consequence on children health such as anaemia [6, 7]. Several malaria control strategies were developed recently, including (i) early case detection and treatment using rapid diagnostic tests (RDT), treatment with more effective antimalarial drugs: artemisinin-based combination therapy (ACT) for uncomplicated malaria cases and pre-referral rectal artesunate for the initial management of complicated malaria, and (ii) intermittent preventive treatment (IPT) for pregnant women and infants among others.

In tropical regions, there are multiple aetiologies to anaemia and intestinal parasites as well as malnutrition can play a major role in anaemia occurrence [8], particularly when malaria prevalence is declining. Thus, exploring the relationship between malaria, intestinal parasites, malnutrition, and anaemia can provide useful information in order to guide public health programs aiming at reduction of child health problems in tropical regions.

This study was aiming (i) to assess the prevalence of malaria parasitaemia, intestinal parasites (IP), anaemia, and malnutrition among children <5 years and (ii) to explore the relationship between malaria, intestinal parasites, malnutrition, and anaemia.

The study was conducted prior to the implementation of an impact study evaluating the impact on malaria burden of a community-based malaria control strategy including effective case management (using RDT and ACT treatment) and IPTc at Lamarame in Senegal, where a demographic surveillance system recently has been established.

## 2. Population and Methods

### 2.1. Study Area and Population

The study was conducted at the Lamarame health post located in the Keur Socé, a rural community, at 17 Km from the city of Kaolack in central Senegal, and 200 Km from Dakar. The Lamarame health post is in the Ndoffane health district. It covers 34 villages located within a radius of 8 Km from the post, with a total population of about 10,000 inhabitants.

Malaria is endemic in this area with a seasonal pattern of transmission peaking during the rainy season (July–November); entomological inoculation rates range from 9 to 12 infective bites/human/year (*Konate et al. unpublished data*). Many programs aiming to reduce child health problems are being promoted in this area. These programs included two yearly vitamin A supplementation, and deworming with Mebendazole, in December and June of each year for children aged 1 to 5 years.

A cross-sectional household survey was carried out at the end of the malaria transmission season, in January 2010, one month after the second round of Mebendazole mass administration campaign.

A two-level random cluster sampling technique was used with a total of 30 clusters randomly selected based on probability proportional size in the villages. The first household was randomly selected in each cluster, followed by the neighbouring household until a minimum cluster target of 23 children was reached. A minimum sample size of 700 children under 5 years of age was calculated using Epi Info software based on an expected prevalence of anaemia of 35%, with alpha = 5% (confidence interval at 95%), power at 90% (beta 10%), and being multiplied by 2 for an design effect.

### 2.2. Data Collection

#### 2.2.1. Structured Questionnaire

A code was given to every child after parents' informed consent. Each eligible child was examined by a physician prior to a biological assessment which included blood and stool samples. The mother was interviewed directly concerning the child's symptoms as well as well as sociodemographic characteristics using a standard questionnaire. Data obtained from physical examination and parents interview were assigned on a case report form (CRF).

#### 2.2.2. Anthropometry

Children were weighed by a trained assistant, without their shoes, using Seca scales; height was measured to the nearest 0.1 cm using a calibrated scale and a sliding head piece. Weight-for-age *Z*-score was used to denote underweight, while height for age *Z*-score was used as an indicator of stunting. The *Z*-scores were calculated based on the median values of the National Centre for Health Statistics (NCHS) reference population, United States.

#### 2.2.3. Biological Assessment

Blood samples were collected using finger prick blood. The first drop was used to prepare thick and thin smears for the diagnosis of malaria. Thick and thin smears were stained with Giemsa and read by a laboratory technician. Malaria was defined as any asexual parasitemia detected on a thick or thin blood smear. Parasitemia was numbered and expressed by number of trophozoites/*μ*L using the following formula: numbered parasites ×8000/200 assuming a white blood cell count of 8000 cells per *μ*L. Absence of malaria parasite in 200 high power ocular fields of the thick film was considered as negative.

The second drop of finger prick blood was drawn into a microcuvette for Hb determination (g/dL) using HemoCue machine (HemoCue Hb 301). Moderate and severe anaemia were defined as hb concentration below 11 g/dL and 8 g/dL, respectively.

Fresh stools samples were collected into wide mouth screw-cap clean containers. Fecal samples were examined for the detection of intestinal parasite using the Ritchie technique. Intestinal parasite was recorded positive by the presence of helminthes and/or protozoans in the faces.

### 2.3. Data Analysis

Data were entered in Excel software and analysed using STATA 10 software. To assess the nutritional status, data were transferred into Epi Info 3.04 d. The *Z*-scores for weight-for-age (underweight) and height-for-age (stunting) were derived using Epinut Anthropometry. Children who had *Z*-scores below −2 standard deviation (SD) of the NCHS reference population median were considered to be malnourished.

For descriptive data, percentage was used to assess the prevalence of each outcome. Proportions were compared using chi-square test or the Fisher exact test (univariate analysis); significance level of the different tests was 0.05 two-sided. A stepwise logistic regression was done for the determination of risk factors possibly associated with anaemia and under-nutrition. The validity of the final models was tested using the Hosmer-Lemeshow goodness of fit test.

## 3. Results

Seven hundred and thirty-six children (393 male and 343 female) aged 6 months–5 years with a mean age of 2.5 ± 1.3 years participated in this study (Table 1).

### 3.1. Prevalence of Malaria, Anaemia, Intestinal Parasites, and Undernutrition

The overall prevalence of *Plasmodium falciparum* was 1.5%. Moderate anaemia and severe anaemia were found in 53.4% and 12.5% of the children, respectively. A total of 193 (26.2%) children were found with at least one intestinal parasite. Parasites species were represented by *Giardia intestinalis* 15.6%, *Entamoeba coli* 10.9, *Hymenolepis nana* 1.9%, *Ascaris lumbricoïdes* 0.4%, *Enterobius vermicularis* 0.4%, and *Trichuris trichiura* 0.1%. Children with two intestinal parasites species represented 3,01% (22 children); one child (0,14%) was coinfected with 3 intestinal parasites species. The overall prevalence of stunting and underweight was 22% and 16.3%, respectively (Table 2).

### 3.2. Factors Associated with Anaemia among Children under Five

Anaemia (Hb < 11 g/dL) was significantly associated with age range from 12 to 23 months (aOR = 3.7 (1.7–7.9)), malaria parasite (aOR = 6.3 (1.5–53.5)), and birth-order higher than 3 (aOR = 1.6 (1.1–3.5)). Stunting was also significantly associated with anaemia in children under 5 years of age (aOR = 2 (1.2–3.1)). There was no statistically significant association between isolated intestinal parasites, such as *Giardia intestinalis*, *Entamoeba coli*, *Hymenolepis nana*, *Enterobius vermicularis*, and *Ascaris lumbricoïdes*, and anaemia. No association was found between anaemia and coinfection with more than one intestinal parasite species (aOR = 0.66 (0.28–1.58)) (Table 3).

### 3.3. Factors Associated with Undernutrition among Children under Five

Multivariate analysis using a logistic regression model showed that stunting was significantly associated with moderate anaemia (OR 1.7 CI 95% (1.1–2.8)), severe anaemia (OR 3.5 (1.9–6.4)), residence zone (OR 3.5 CI 95% (1.6–8.1)), and birth order >3 (OR 2.9 (1.3–6.6)). There was no statistically significant association between malaria and stunting (OR 1.2 (0.3–0.5)) as well as isolated intestinal parasites such *Giardia intestinalis*, *Entamoeba coli*, *Hymenolepis nana*, *Enterobius vermicularis*, and *Ascaris lumbricoïdes* (Table 4).

Factors associated with underweight were represented by moderate anaemia (aOR 1.7 (1.1–2.8)), severe anaemia (aOR 4.4 (2.3–8.4)), and age range from 24 to 35 months (aOR 8.9 (1.2–70.6)) (Table 5).

## 4. Discussion

Malaria, anaemia, intestinal parasitic infections, and malnutrition remain serious public health problems especially in developing countries [9, 10]. We assessed the magnitude of malaria, intestinal parasites carriage, anaemia, and under-nutrition.

Our study conducted in a malaria endemic area showed an unexpectedly low prevalence of *P. falciparum* infections (1.5%), while a high level of anaemia was observed. Despite the low prevalence of malaria parasitoemia, anaemia was more frequent in children with *P. falciparum* infections (OR 6.3). It, thus, appears that malaria is still closely associated with anaemia in this area, even in the context of the decline of malaria prevalence. A survey conducted in January 2009 (one year before) in a neighbouring area (Kaolack: 17 Km from the Lamara me health post) showed a higher prevalence of malaria (7.2%) [11]. These results suggest the decrease of malaria prevalence in this part of Senegal as well as in other areas (*National Malaria Control program annual report 2009*). Scaling up antimalarial strategies such as early case detection and treatment, intermittent preventive treatment as well as long-lasting insecticide treated nets may even contribute to malaria elimination in this area and significantly reduce anaemia as a public health problem in rural Senegalese areas with similar disease patterns. Children with a birth order greater than three had an increased risk of developing anaemia. In other studies, high family size was associated with anaemia and undernutrition of children [12].

The study showed a high prevalence of intestinal parasite carriage (26.2%); *Giardia intestinalis* was the most frequent parasite (15.6%). The observed prevalence of *Giardia* in this study is consistent with those described in other countries [13, 14]. The deleterious effect of *Giardia intestinalis* on growth and health of children has been shown by several studies [15]. Although we did not find any statistically significant association between *Giardia intestinalis* and anaemia, as well as malnutrition, this parasite is known for its ability to induce diarrhoea [16] and malabsorption syndrome, and it can lead to protein energy malnutrition, vitamin A deficiency, iron deficiency anaemia, and vitamin B12 deficiency anaemia [17]. Mass deworming programme is being promoted at the Lamarame health post to reduce intestinal worms carriage. In December 2009, a large scale of mass administration of mebendazole took place at the Lamarame health post with an estimated coverage of 95% in children aged 1 to 5 years (*Lamarame health post report-unpublished data*). This can explain the low level of prevalence of helminthic infections. While ensuring an effective action against helminthic infections, mass administration of mebendazole does not act against protozoan infections. Albendazole can have an effective action against both protozoan and helminthic infections and can be a suitable molecule in mass deworming programs, particularly in areas where Giardia is frequent. Thus, replacing mebendazole by albendazole during a mass deworming programme could further reduce the frequency of helminthic infections as well as protozoan infections and, consequently, the occurrence of diarrheal disease and malabsorption syndrome.

Malnutrition remains frequent in children under 5 years with a prevalence of 22% of stunting and 16.3% of underweight. In 2005, the fourth demographic and population health survey found an average rate of stunting in rural areas of 20.6%, and the prevalence of underweight was 21.6% [18]. Our data have confirmed the classical association between anaemia and malnutrition [19]. Indeed, an inadequate intake of macro- and micronutrients, or intestinal malabsorption induced or increased by intestinal parasites infections, can play through iron and folate deficiency a well-documented role in chronic anaemia pathophysiology [8].

*Study Limitations*. This study, conducted in a rural Senegalese area, has provided useful data for the control of some frequent tropical diseases. However, sociocultural and behavioural factors which may have a relationship with the occurrence of anaemia and/or malnutrition have not been evaluated in this study, in particular the intake of macro- and micronutrients and the patterns of weaning and supplementary feeding.

## 5. Conclusion

Anaemia, intestinal parasites, and malnutrition are still frequent in the area of Lamarame, while malaria is declining in this part of Senegal. Despite the decrease of malaria in this area, malaria is still a contributing factor to anaemia in this area, due to the close association between malaria and anaemia. The combination of several antimalarial interventions may contribute to eliminating malaria in this region and reducing the occurrence of anaemia among children.

## Figures and Tables

**Table 1:** Sociodemographic characteristics of participating children under 5 years at Lamarame (*n* = 736).

Sociodemographic variables	Number	Percentage	CI 95%
Age (months)			
<12	42	5.7	(4.1–7.7)
(12–23)	239	32.5	(28.5–36.9)
(24–35)	197	26.8	(23.1–30.7)
(36–47)	137	18.6	(15.6–22)
(48–60)	121	16.4	(13.6–19.6)
Gender			
Male	393	53.4	(48.2–58.9)
Female	343	46.6	(41.8–51.8)
Birth order of children			
(1–3)	393	53.4	(48.2–58.9)
(4-5)	340	46.2	(41.4–51.4)
Number of children in household			
(1–3)	309	42	(37.4–46.9)
(4-5)	216	29.4	(25.6–33.5)
>5	202	27.4	(23.8–31.5)
Missing	9	1.2	(0.5–2.3)
Residence zone			
Lamarame	116	15.8	(13–18.9)
Others villages	620	84.2	(77.7–91.1)

**Table 2:** Prevalence of malaria, anaemia, intestinal parasites, and undernutrition among children under 5 years at Lamarame health post (*n* = 736).

Variables	Number	Percentage	CI 95%
Malaria parasite			
Yes	11	1.5	(0.7–2.6)
No	721	98	(90.9–100)
Anaemia			
Mean haemoglobin	736	10.1 ± 1.9	
Moderate anaemia (Hb < 11)	393	53.4	(48.2–58.9)
Severe Anaemia (Hb < 8)	92	12.5	(10–15.3)
Intestinal parasite prevalence			
Children with at least one intestinal parasite	193	26.2	(22.6–30.2)
Parasite species			
*Giardia intestinalis *	115	15.6	(12.9–18.7)
*Entamoeba coli *	80	10.9	(8.6–13.5)
*Hymenolepis nana *	14	1.9	(1–3.2)
*Ascaris lumbricoïdes *	3	0.4	(0.08–1.2)
*Enterobius vermicularis *	3	0.4	(0.08–1.2)
*Trichuris trichiura *	1	0.1	(0.003–0.7)
Undernutrition			
Stunting (HAZ < −2 SD)	161	22	(18.6–25.5)
Underweight (WAZ < −2 SD)	120	16.3	(13.5–19.5)

**Table 3:** Factors associated with anaemia among children under five at Lamarame health post.

Variables	Anaemia (Hb < 11 g/dL)		
	Number (%)	aOR∗ (95% CI)	*P* value
Age (months)			
<12	19 (45.2)	1	
12–23	199 (83.3)	3.7 (1.7–7.9)	0.001
24–35	136 (69)	1.6 (0.7–3.5)	0.20
36–47	83 (60.6)	1.1 (0.5–2.4)	0.75
48–60	54 (44.6)	0.6 (0.3–1.4)	0.26
Gender			
Male	264 (67.2)	1	
Female	227 (66.2)	0.8 (0.6–1.2)	0.37
Birth order			
(1–3)	254 (64.6)	1	
(4-5)	235 (69.1)	1.6 (1.1–3.5)	0.04
Number of children within household			
(1–3)	213 (68.9)	1	
(4-5)	133 (61.6)	0.5 (0.3–0.8)	0.03
>5	137 (67.8)	0.6 (0.3–1.3)	0.26
Malaria parasite			
No	479 (66.4)	1	
Yes	10 (90.9)	6.3 (1.5–53.5)	0.03
Stunting			
No	352 (62.4)	1	
Yes	132 (81.9)	2 (1.2–3.1)	0.004
Intestinal parasite			
No	369 (68.7)	1	
*Giardia intestinalis *	73 (63.5)	0.9 (0.6–1.4)	0.75
*Entamoeba coli *	49 (61.2)	1.1 (0.6–1.9)	0.67
*Hymenolepis nana *	10 (71.4)	1.8 (0.5–6.7)	0.35
*Enterobius vermicularis *	1 (33.3)	0.2 (0.02–2.7)	0.28
*Ascaris lumbricoïdes *	1 (33.3)	0.2 (0.01–2.7)	0.28
Residence zone			
Health post	55 (47.4)	1	
Other villages	436 (70.3)	2.1 (1.3–3.4)	0.001

^*^Adjusted odds ratio. Goodness of fit test: Hosmer-Lemeshow *χ*² (8df) = 5.49, *P* = 0.70.

**Table 4:** Factors associated with stunting among children less than five years at Lamarame health post.

Variables	Stunting (HAZ)		
	Number (%)	aOR∗ (95% CI)	*P* value
Age (months)			
<12	1 (2.4)	1	
12–23	64 (26.8)	7.4 (1–57.3)	0.05
24–35	49 (24.9)	7.5 (1–58.1)	0.05
36–47	29 (21.1)	6.6 (0.8–52.3)	0.07
48–60	18 (14.9)	5 (0.6–41)	0.13
Gender			
Male	88 (22.4)	1	
Female	73 (21.3)	0.9 (0.6–1.4)	0.82
Birth order			
(1–3)	82 (20.8)	1	
(4-5)	79 (23.2)	2.9 (1.3–6.6)	0.01
Number of children within household			
(1–3)	75 (24.2)	1	
(4-5)	43 (19.9)	0.4 (0.2–0.8)	0.01
>5	42 (20.8)	0.3 (0.1–0.7)	0.01
Malaria parasite			
No	157 (21.8)	1	
Yes	3 (27.3)	1.2 (0.3–5)	0.75
Intestinal parasite			
No	123 (22.9)	1	
*Giardia intestinalis *	21 (18.3)	0.9 (0.5–1.6)	0.73
*Entamoeba coli *	17 (21.2)	1.2 (0.7–2.3)	0.45
*Hymenolepis nana *	3 (21.4)	1.3 (0.3–5.4)	0.69
*Enterobius vermicularis *	00	—	
*Ascaris lumbricoides *	00	—	
Residence zone			
Health post	7 (6)	1	
Other villages	154 (24.8)	3.6 (1.6–8.1)	0.002
Anaemia			
No	29 (11.8)	1	
Moderate anaemia (Hb < 11 g/dL)	93 (23.6)	1.7 (1.1–2.8)	0.02
Severe anaemia (Hb < 8 g/dL)	39 (39.8)	3.5 (1.9–6.4)	0.000

^*^Adjusted odds ratio. Goodness of fit test: the Hosmer-Lemeshow *χ*² (8df) = 7.45; *P* = 0.48.

**Table 5:** Factors associated with underweight among children less than five years at Lamarame health post.

Variables	Underweight (WAZ)		
	Number (%)	aOR∗ (95% CI)	*P* value
Age (months)			
<12	1 (2.4)	1	
12–23	38 (15.9)	4.9 (0.6–39.5)	0.12
24–35	43 (21.8)	8.9 (1.2–70.6)	0.03
36–47	22 (16)	6.4 (0.8–52)	0.08
48–60	16 (13.2)	6.2 (0.7–50.8)	0.09
Gender			
Male	64 (16.2)	1	
Female	56 (16.3)	1.1 (0.7–1.6)	0.7
Birth order			
(1–3)	66 (16.8)	1	
(4-5)	54 (15.8)	1.8 (0.8–4.2)	0.15
Number of children within household			
(1–3)	58 (18.7)	1	
(4-5)	33 (18.3)	0.5 (0.2–1.2)	0.16
>5	28 (13.7)	0.4 (0.1–1.1)	0.09
Malaria parasite			
No	117 (16.2)	1	
Yes	2 (18.2)	0.9 (0.2–4.8)	0.9
Intestinal parasite			
No	98 (18.2)	1	
*Giardia intestinalis *	11 (9.6)	0.6 (0.3–1.1)	0.09
*Entamoeba coli *	8 (10)	0.6 (0.3–1.3)	0.19
*Hymenolepis nana *	2 (14.3)	1.3 (0.2–6.7)	0.73
*Enterobius vermicularis *	1 (33.3)	4.4 (0.3–61.1)	0.26
*Ascaris lumbricoïdes *	00	—	—
Residence zone			
Health post	9 (7.7)	1	
Other villages	111 (17.9)	0.6 (0.3–1.4)	0.27
Anaemia			
No	24 (9.8)	1	
Moderate anaemia (Hb < 11 g/dL)	63 (16)	1.7 (1.1–2.9)	0.04
Severe anaemia (Hb < 8 g/dL)	33 (33.7)	4.4 (2.3–8.4)	0.000

^*^aOR: adjusted odds ratio. Goodness of fit test: the Hosmer-lemeshow *χ*² (8df) 3.35; *P* = 0.91.
